# What would John Maynard Keynes say about contemporary oral health? Reflections on preferences, social conventions, and marketisation

**DOI:** 10.3389/froh.2026.1986109

**Published:** 2026-10-22

**Authors:** Demetrio Lamloum, Stefan Listl

**Affiliations:** 1 Department of Restorative, Preventive and Pediatric Dentistry, School of Dental Medicine, University of Bern, Bern, Switzerland; 2 Section for Oral Health, Heidelberg Institute of Global Health (HIGH), Heidelberg University Hospital, Heidelberg, Germany

**Keywords:** commercial determinants of health, health policy, Keynes, oral health, public health

## Introduction

In 2026, eighty years after the death of John Maynard Keynes, his ideas continue to be widely invoked in public debate. Contemporary discussions frequently reduce Keynesian thought to questions of public debt, government spending, and countercyclical interventions. Yet Keynes's intellectual legacy extends far beyond fiscal policy and reaches areas that seem out of scope, such as dentistry. We encountered this connection while researching oral healthcare financing in the European Union, where the gap between what people need and what markets provide seemed to require a theoretical frame beyond standard welfare economics. Keynes's reflections on uncertainty, conventions, socially generated desires, and the distinction between absolute and relative needs offer important insights into how oral healthcare preferences are formed in increasingly commercialised environments.

Oral healthcare occupies a paradoxical position in contemporary public health. Despite oral diseases being among the most prevalent non-communicable conditions globally, they are frequently framed as largely preventable through individual behaviour (1). This framing mirrors broader economic and policy discourses that emphasise consumer choice as the primary driver of social outcomes. Individuals are increasingly positioned as consumers navigating markets for oral hygiene products, preventive services, or aesthetic treatments. The underlying assumption is that individuals possess stable preferences and sufficient information to make rational choices that optimise their health. The evidence suggests that these conditions are rarely met. Persistent and widening social gradients in oral health outcomes are difficult to reconcile with this assumption (2, 3). Moreover, the expanding role of commercial actors, ranging from idealised beauty models to cosmetic dentistry markets, raises questions about how oral healthcare preferences are shaped and whose interests drive such trends (4).

Our argument is written from the perspective of high-income countries, mainly in Europe, where dental care is financed through a mix of public and private sources and where a large private market for elective and aesthetic treatment exists. Therefore, it does not apply to low- and middle-income countries which carry different burdens (*e.g.,* absence of basic care and out-of-pocket payment, and financial hardship) (5, 6).

## Transferring Keynesian concepts to oral health

Keynes did not write about health or dentistry, and his analysis of uncertainty and conventions was developed for investment decisions. We use his concepts as analytical lenses. Three features make the transfer reasonable. First, Keynes's essay Economic Possibilities for our Grandchildren (1930), where he distinguishes between absolute needs and relative needs (7, p. 326); second, Keynes's central idea is that many decisions are made under fundamental uncertainty, where probabilities cannot be calculated and people therefore rely on conventions. This links to Arrow's 1963 paper, widely regarded as a founding text in health economics, which showed that uncertainty about the incidence of illness and the effects of treatment, together with unequal information between providers and patients, are defining features of the market for medical care (7). Arrow's uncertainty is closer to measurable risk than Keynes's, but both analyses reach the same point, where outcomes cannot be judged in advance, people rely on others' judgement; third, Keynes's policy conclusion, that the state should act where private markets cannot be relied on, addresses directly the topic of public investment in oral healthcare.

## Absolute and relative needs in oral health

In defining needs in the aforementioned essay, Keynes distinguishes absolute needs, which are felt regardless of social comparison, from relative needs, which emerge through comparison, status competition, and the desire for distinction. The same idea appears in Veblen's conspicuous consumption, in which goods are consumed to display wealth and rank above others (8), and in Hirsch's positional goods, whose value depends on the position of the user relative to others and falls as more people obtain them (9). This distinction is highly relevant to oral health, and should not be interpreted narrowly. Contemporary understandings of health, particularly those influenced by the WHO definition, recognise that health includes psychological well-being, social participation, communication, and self-esteem (10). Oral health matters legitimately for confidence, employability, social interaction, and quality of life. The problem emerges when these dimensions of well-being become increasingly colonised by market forces that continuously redefine them. Teeth that meet clinical standards of health may still be perceived as imperfect if they are not sufficiently white, straight, youthful, or aesthetically desirable. The market can then expand not by addressing unmet health needs but by amplifying comparative dissatisfaction, turning well-being into an open-ended consumer project in which the standard for “acceptable” teeth is repeatedly moved.

The boundary between absolute and relative needs is not sharp, but it can be approached in practice. Table 1 proposes a working typology with four categories. Three questions help place a treatment. Is there disease, pain, or loss of function that can be assessed clinically? Would the need remain if everyone else's teeth looked the same, which is the Keynesian test of whether satisfaction depends on comparison? Does the demand rise when the average standard of appearance rises? Dentistry already has an instrument that draws a similar distinction: the Index of Orthodontic Treatment Need separates a Dental Health Component, which captures clinically assessed need, from an Aesthetic Component, which captures perceived aesthetic impairment (11). A similar logic could be applied to other elective treatments.

**Table 1:** A working typology of absolute and relative oral health needs.

Type of need	Definition/distinguishing feature	Examples	What generates the need?	Public financing implication
Absolute (biological)	Pain or risk of disease, identifiable independently of social comparison	Caries, periodontitis, infection, pain	Biological disease or risk	Strong basis for public coverage
Absolute (functional)	Need arising from clinically relevant impairment of oral function or participation	Replacement of missing teeth affecting chewing or severe malocclusion; cleft care	Loss of function, communication or participation	Basis for public coverage, using defined need criteria
Mixed/context-dependent	Need combines an objective oral condition with psychosocial effects; the importance varies across individuals and contexts	Visible fluorosis or traumatic discolouration; moderate malocclusion	Clinical condition + individual psychosocial and social context	Potential public coverage. Requires case-by-case assessment and validated measures
Relative need	Need primarily generated by comparison with others and changing social standards rather than disease or functional impairment	Whitening of healthy teeth; veneers on sound teeth; minor orthodontic irregularities	Social comparison, status and changing norms of appearance	Limited case for collective financing; primarily private financing, with consumer protection and regulation of advertising

## Preferences, conventions, and animal spirits in oral health behaviour

In The General Theory of Employment, Interest and Money (1936), Keynes explains that decisions are made in contexts where future outcomes cannot be precisely assessed (12). Under such conditions, individuals rely on habits, conventions, and shared beliefs rather than strict rational optimisation. Investors, in his account, spend their energy trying to guess “what average opinion expects the average opinion to be” (13, p. 156), much economic activity, investment in particular, is driven less by calculated probabilities than by what Keynes termed “animal spirits”, a spontaneous urge to act rather than a weighted comparison of costs and benefits (13, p. 161). This insight challenges the neoclassical assumption of stable, exogenous preferences driven by an individual rational utility-maximising agent (13). Preferences are instead formed and revised within social contexts that provide cues about what is normal, desirable, and acceptable.

Following this logic, demand is not simply the aggregation of individual utilities, but a social phenomenon shaped by collective expectations (14). These conventions are reinforced through institutions, media, and market practices. Applied to oral health, this framework challenges the assumption that individuals freely choose healthy or unhealthy behaviours on the basis of objective information. Oral health behaviours are embedded in conventions related to responsibility, risk, appearance, and morality, which are increasingly influenced by commercial interests (15).

Historically, improvements in oral health were largely driven by population-level interventions, including water fluoridation, improved nutrition, and expanded access to dental services (16, 17). The relative weight of these factors is debated, and many experts consider fluoride toothpaste, a commercial product whose use was supported by public health endorsement, to be the main reason for the decline of dental caries (18). Oral health was long conceptualised as a public good, influenced by social, environmental, political, and economic conditions, and oral public health frameworks emphasised structural prevention and recognised oral health inequalities as reflections of broader social inequalities (19). In recent decades, oral health has increasingly been framed as a matter of individual responsibility, in line with broader neoliberal trends in health governance in which responsibility is devolved from collective institutions to individuals (20). This shift has contributed to greater patient awareness and may have encouraged healthier behaviours, potentially leading to improved population health outcomes. At the same time, it has also opened the door to adverse outcomes, such as the overconsumption of dental services. The growth of cosmetic dentistry illustrates how markets increasingly redefine health norms. One commercial market research firm values the global cosmetic dentistry market at about USD 32 billion in 2025 and forecasts nearly USD 100 billion by 2034 (21); other firms estimate it at about USD 28–29 billion in 2025 and forecast about USD 60–66 billion by 2034–2035 (22, 23). These are industry estimates whose methods cannot be verified, and the size of the disagreement shows how uncertain they are. Nevertheless, they illustrate how whitening, aligners, and other aesthetic treatments are increasingly framed as health necessities (24). In Keynesian terms, part of what the cosmetic dentistry industry sells responds to relative rather than absolute needs, and the redefinition of social conventions expands demand by reshaping what counts as “essential” oral health (25).

## Provider incentives and corporate ownership

Preferences can also be shaped by the supply side. As introduced before, patients' judgements also rely on providers' recommendations. Supplier-induced demand in dentistry has been documented. In the United Kingdom, Birch found patterns of treatment consistent with supplier inducement under a fixed-fee system (26); in the Netherlands, an experiment that liberalised dental fees in 2012 was followed by price increases and was reversed after one year (27). A more recent force is the purchase of dental practices by corporate groups and private equity funds. In the United States, the proportion of dentists affiliated with a dental service organisation increased from 7.4% in 2015 to 16.1% in 2024 and reached 26.5% among dentists in their first ten years of practice, and the share affiliated with private equity almost doubled between 2015 and 2021, from 6.6% to 12.8% (28, 29). Dental offices acquired by private equity firms tended to shift from diagnostic and preventive procedures to generally higher reimbursement restorative, specialty, and surgical procedures (30). In the European Union, dental chains are legally permitted in 24 of 26 countries surveyed, non-dentists may own practices in 22, and 11 countries reported an increase in dentists working in chains since 2018 (31). Commercial pressures can conflict with professional obligations and are associated with concerns about overtreatment (24). In Keynesian terms, investor ownership brings the expectations of financial markets, and its interest in growth and returns, into dental clinics. This is a strong driver towards marketisation, and it operates regardless of how well-informed patients are.

## Commercial determinants and the marketisation of oral health needs

Commercial determinants play a central role in shaping oral health-related preferences also outside the dental clinic. Oral hygiene products, dental services, and food and beverage items are marketed through narratives that link consumption to health, responsibility, and social acceptance. These practices go beyond information and actively construct preferences and expectations. Despite strong evidence linking sugar consumption to dental caries, sugar-rich foods and beverages are widely marketed as compatible with healthy lifestyles, particularly to children and adolescents (32). Such marketing contributes to conventions that normalise high sugar consumption and undermine individual efforts to reduce risk (33).

As oral health becomes increasingly governed through markets, commercial solutions expand while structural interventions are de-prioritised. Scarce public funding does not cause marketisation by itself, but the two reinforce each other. When public budgets are cut, services that are only partly covered, such as dental care, are easy to reduce, and private provision fills the gap. As private spending grows, so does the share of care that is organised around market demand rather than need.

A Keynesian reading would caution against relying on market-driven conventions to achieve equitable oral health outcomes, since it recognises that preferences and behaviours are socially produced rather than purely individual. Policy approaches informed by this framework would therefore emphasise upstream interventions, including regulation of harmful marketing and fiscal measures targeting risk factors (*i.e*., the sugar industry). Keynes's main policy idea was that when private markets cannot be relied on to produce what society needs, the state should act. *In The End of Laissez-Faire*. “The important thing for Government is not to do things which individuals are doing already, and to do them a little better or a little worse; but to do those things which at present are not done at all” (35, p. 291). This suggests three priorities for public action. First, workforce planning based on need rather than market demand. Left to the market, dentists concentrate where paying demand is highest, which is often in cities. Needs-based planning models can estimate the numbers to meet population needs (34). Second, public dental services for groups the market serves poorly. Children, older people in residential care, people with disabilities, and low-income households are not profitable markets for most private providers. Salaried or publicly contracted services, with payment systems that do not reward volume, such as capitation or blended payment, can provide prevention and basic care for these groups (35). Third, protecting prevention budgets. The returns to prevention come over many years, which makes prevention an easy target when public budgets are cut. A Keynesian approach would treat prevention as public investment rather than current spending, and would protect it, or increase it, precisely when private demand and public revenues fall.

## Conclusion

The contemporary framing of oral health as an individual choice reflects broader economic narratives emphasising consumer sovereignty. Keynesian economic theory suggests that oral health preferences are socially constructed through conventions and commercial influences. Advertising, marketing, and market expansion contribute to redefining oral health, shifting responsibility onto individuals while obscuring structural and commercial determinants. The same forces act on the supply side: fee-for-service payment and the growing ownership of dental practices by corporate groups and private equity funds bring financial incentives into clinical decisions, regardless of how well informed patients are Recognising oral health as a socially embedded good supports a renewed emphasis on collective action, regulation, and public investment to reduce oral health inequalities. Following Keynes, the state should invest where markets do not provide: in workforce planning based on need, in public dental services for groups the market serves poorly, and in prevention budgets protected from cuts when money is tight. Keynes once hoped that economists could become “humble, competent people, on a level with dentists” (7, p. 332), (36), a modest professional ideal grounded in competence rather than persuasion. Oral health is currently moving in the opposite direction, shaped by commercial incentives that forge demand, redefine what is considered essential, and turn health into an open-ended project of consumption. Both economics and dentistry, in this sense, serve society best when they remain focused on needs rather than on manufactured demand.
